# Enhancing Lignin Dissolution and Extraction: The Effect of Surfactants

**DOI:** 10.3390/polym13050714

**Published:** 2021-02-26

**Authors:** Elodie Melro, Artur J. M. Valente, Filipe E. Antunes, Anabela Romano, Bruno Medronho

**Affiliations:** 1 Department of Chemistry, University of Coimbra, CQC, Rua Larga, 3004-535 Coimbra, Portugal; avalente@ci.uc.pt (A.J.M.V.); fcea@ci.uc.pt (F.E.A.); 2 MED—Mediterranean Institute for Agriculture, Environment and Development, Faculdade de Ciências e Tecnologia, Campus de Gambelas, Universidade do Algarve, Ed. 8, 8005-139 Faro, Portugal; aromano@ualg.pt (A.R.); bfmedronho@ualg.pt (B.M.); 3 FSCN, Surface and Colloid Engineering, Mid Sweden University, SE-851 70 Sundsvall, Sweden

**Keywords:** dissolution, extraction, lignin, lignocellulosic waste, maritime pine, surfactants

## Abstract

The dissolution and extraction of lignin from biomass represents a great challenge due to the complex structure of this natural phenolic biopolymer. In this work, several surfactants (i.e., non-ionic, anionic, and cationic) were used as additives to enhance the dissolution efficiency of model lignin (kraft) and to boost lignin extraction from pine sawdust residues. To the best of our knowledge, cationic surfactants have never been systematically used for lignin dissolution. It was found that ca. 20 wt.% of kraft lignin is completely solubilized using 1 mol L^−1^ octyltrimethylammonium bromide aqueous solution. A remarkable dissolution efficiency was also obtained using 0.5 mol L^−1^ polysorbate 20. Furthermore, all surfactants used increased the lignin extraction with formic acid, even at low concentrations, such as 0.01 and 0.1 mol L^−1^. Higher concentrations of cationic surfactants improve the extraction yield but the purity of extracted lignin decreases.

## 1. Introduction

The interaction of surfactants with macromolecules, such as proteins, dates from the early 1950s [1]. This research focusing on these interactions was mainly triggered by their biological relevance. Later, synthetic polymers with well-defined characteristics appeared, and research was further extended to other polymer–surfactant systems. Due to their uses in different applications, such as cosmetics, paints, foods, detergents, pesticides, and pharmaceutics, polymer–surfactant systems have received a lot of attention in the last several decades [2,3]. The combination of polymers and surfactants can offer myriad effects, such as controlling interfacial properties, network formation, and rich phase behavior. The latter effect is particularly relevant in the context of the solubilization of water-insoluble polymers [4]. Lignin is an example of a polymer that is insoluble in water and many other traditional solvents. This complex polyphenolic biopolymer is considered the second most available macromolecule on Earth and works as a “natural glue,” holding together cellulose and hemicellulose in the fibers of plant cell walls [5]. Lignin possesses great potential to obtain value-added compounds, but its uses are still scarce; lignin is mainly burnt for energy generation purposes [6]. As mentioned, lignin dissolution is complicated but frequently required as a first step for several applications. Despite the dissolution limitations, several solvent systems have been successfully developed [7]. In some cases, different additives have proven to enhance lignin dissolution [8]. In this respect, surfactants are typically good candidates to improve polymer solubilization, since their association with macromolecules can turn them more hydrophilic, decrease hydrophobic interactions, introduce charges, etc. Surfactant (and polymer) properties, such as hydrophobicity, chain flexibility, charge density, and ionic strength, are expected to affect the binding and aggregation phenomena, and even control the dissolution process [9]. Despite being appealing, surfactant application in lignin dissolution has been surprisingly scarce. Among the few reports, Xu et al. have demonstrated the enhancement of lignin dissolution in aqueous-based systems containing the nonionic surfactant tween 80 [10]. In another work, Norgren et al. have used bile acid salts (a special class of biological surfactants) to successfully increase the solubility and colloidal stability of kraft lignin [11]. The search for efficient lignin extraction methods is a topic of great scientific and industrial interest. Lignin is an effective source of biomass, with enzymatic hydrolysis being key to its use [12,13]. In lignocellulosic biomass processing, non-ionic surfactants are typically used to enhance lignin extraction [14,15]. These surfactants play the role of additives in the soda pulping of bagasse [16] and increase the yield of kraft lignin removal in the precipitation step [17]. Non-charged surfactants, mainly the tween-type, are important in enzymatic hydrolysis, and thus are widely used for the conversion of lignocellulosic biomass into bioethanol. Other surfactant systems have been reported to assist the enzymatic hydrolysis in different lignocellulosic biomass matrixes [18,19,20,21,22,23,24,25,26]. During these processes, lignin acts as a physical barrier to the enzymes and can even absorb them via strong interactions (hydrophobic, electrostatic, and hydrogen-bonding) [27,28]. Surfactants facilitate the access of the enzyme to cellulose by removing the lignin [18]. The presence of surfactants not only may increase the reaction yield but also allows a substantial reduction in the amount of needed enzyme (up to ca. 50%), while maintaining a superior yield [23]. The surfactant efficiency in lignin removal can be influenced by several factors, such as its critical micelle concentration (CMC) [22], the chemical and molar mass properties of lignin [8], hydrolysis conditions [28], and substrate type [29].

**Figure 1:**
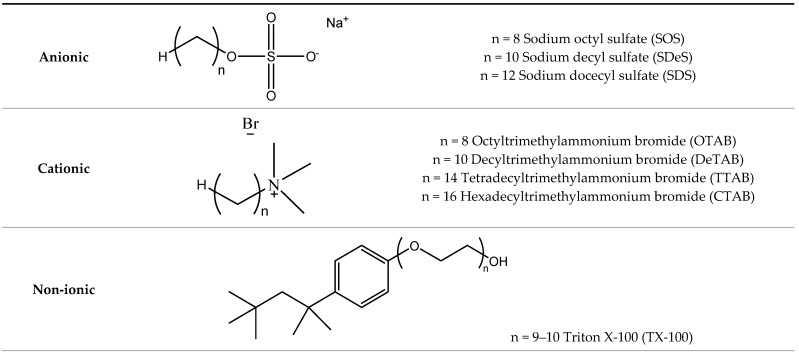

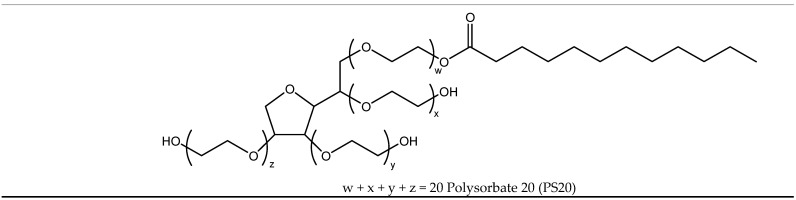
Molecular structures of the surfactants used in this work.

To our knowledge, charged surfactants have never been used to dissolve lignin. Thorough studies comparing several classes of surfactants are lacking. Therefore, in the first part of this study, different cationic and anionic surfactants (Figure 1) were used in an aqueous solution to dissolve model kraft lignin. Non-ionic surfactants were also tested for comparison. In this first part, the interaction between lignin and a cationic surfactant was also followed by electrical conductance, which has not been reported before. In the second part of this study, the most promising surfactants used for model lignin dissolution were tested as potential additives to improve the yield of lignin extraction from lignocellulosic biomass (maritime pine sawdust).

## 2. Materials and Methods

### 2.1. Materials

Kraft lignin, sodium dodecyl sulfate (SDS), decyltrimethylammonium bromide (DeTAB), hexadecyltrimethylammonium bromide (CTAB), and Triton X-100 (TX-100) were purchased from Merck (Algés, Portugal). Sodium octyl sulfate (SOS), sodium decyl sulfate (SDeS), and polysorbate 20 (PS20) were acquired from Fluka (Bunchs, Switzerland). Octyltrimethylammonium bromide (OTAB) was obtained from Tokyo Kasei Kogyo Co., Ltd. (Tokyo, Japan) and tetradecyltrimethylammonium bromide (TTAB) was purchased from LabKemi (Stockholm, Sweden). Sodium hydroxide (NaOH) was purchased from José Manuel Gomes dos Santos, Lda., (Porto, Portugal) and formic acid from Merck (Algés, Portugal). All the chemicals were used as received. Maritime pine (*Pinus pinaster* Ait.) sawdust was received from Valco - Madeiras e Derivados, S.A. (Leiria, Portugal) as a kind gift and was dried before further use.

### 2.2. Conductivity Measurements

The surfactant–lignin interactions (DeTAB surfactant was selected) were probed by electrical conductometry of different solutions using a Wayne–Kerr model 4265 Automatic LCR meter (Wayne Kerr Electronics Ltd, Bognor Regis, United Kingdom), at 1 kHz and 25.00 °C. A dip-type conductivity cell, with a cell constant of 0.1002 cm^−1^ and an uncertainty of 0.02%, was used [30]. The temperature was kept constant by using a HAAKE Phoenix II P2 thermostat bath (Thermo Fisher Scientific, Waltham, MA, US). In a typical experiment, 30 mL of water or lignin solution was placed in the conductivity cell and, after the thermostabilizing, aliquots of the DeTAB solution (1.5 mol L^−1^) were added stepwise with a micropipette (50 μL). The experimental electrical specific conductance, κ_exp_, was measured after each addition of surfactant and computed using an in-house-made software. The specific electrical conductance, κ, was obtained after subtracting the specific conductance of the solvent.

### 2.3. Dissolution Efficiency

The dissolution tests were performed with 20 wt.% model kraft lignin using different concentrations of surfactant. The solutions were stirred at 1000 rpm using a magnetic stirrer for 24 h at 20–25 °C. The dispersions were centrifuged at 14,000 rpm for 1 h and the supernatant was removed and diluted with 1 wt.% NaOH aqueous solution. The dissolved lignin content was obtained by measuring the absorbance of the diluted solution, at 288 nm, using a UV–VIS spectrophotometer, Shimadzu UV-2450 (Shimadzu Corporation, Tokyo, Japan). The results presented are the average of three samples. The dissolution efficiency was further evaluated by optical microscopy, Linkam LTS 120 microscope (Linkam Scientific Instruments Ltd., Tardworth, United Kingdom), and a QImaging station,-QICAM Fast 1394 Digital Camera (Teledyne Photometrics, Tucson, AZ, US) was used for image acquisition.

### 2.4. Lignin Extraction and Purity Determination

**Figure 2:**
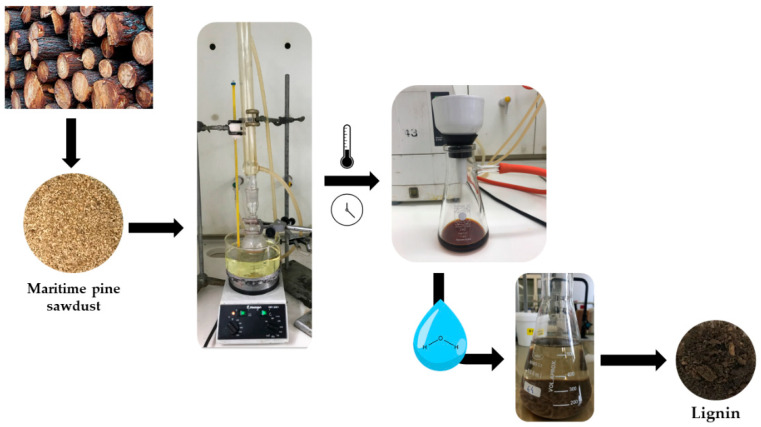
Experimental scheme for lignin extraction.

Lignin was extracted from pine sawdust (1 g) using 10 mL formic acid and different concentrations of surfactant. The mixtures were kept at 160 °C for 2 h in a paraffin bath. Afterward, the liquid fraction was separated by vacuum filtration, and 500 mL of deionized water was added to trigger lignin precipitation. After centrifugation, the collected solid was dried with a freeze dryer. An experimental scheme of the lignin extraction can be observed in Figure 2. The purity of the recovered lignin was determined by the sum of the acid-insoluble and acid-soluble lignin, using the procedures LAP-003 and LAP-004, respectively, by the National Renewable Energy Laboratory [31,32]. Briefly, ca. 0.1–0.3 g of the extracted material was hydrolyzed with 3 mL of 72% H_2_SO_4_ for 1 h at 30 °C. Then, the mixture was diluted with deionized water to 4% H_2_SO_4_ and placed in the oven at 121 °C for 1 h. The solution was cooled and vacuum-filtered with previously dried and weighted filters. The total solid content was used to determine the insoluble lignin fraction. The filtrate obtained was used to estimate the acid-soluble lignin content by measuring the absorbance at 205 nm in a UV–VIS spectrophotometer, Shimadzu UV-2450 (Shimadzu Corporation, Tokyo, Japan). All analyses (extraction and purity determination) were carried out in duplicates.

## 3. Results and Discussion

### 3.1. Lignin Dissolution

**Figure 3:**
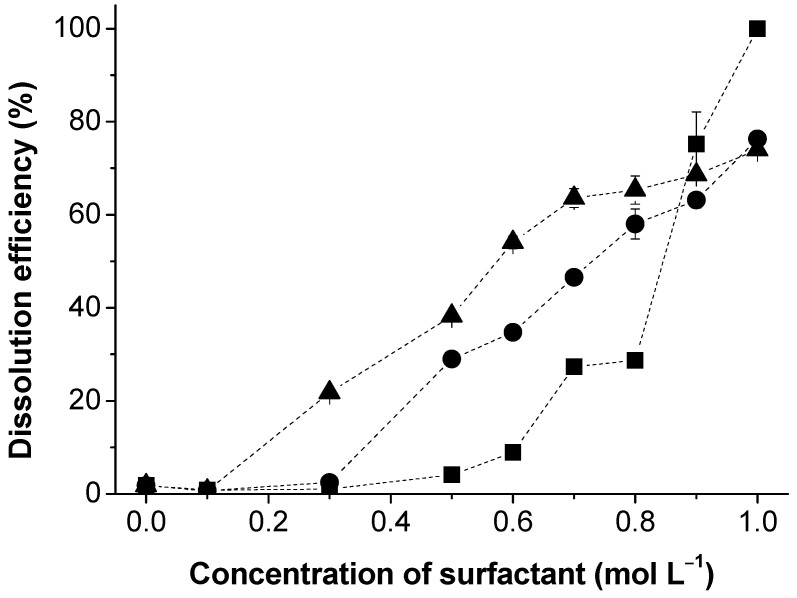
Dissolution efficiency of kraft lignin in water as a function of cationic surfactants: octyltrimethylammonium bromide (OTAB) (squares), decyltrimethylammonium bromide (DeTAB) (circles), and tetradecyltrimethylammonium bromide (TTAB) (triangles).

In the first part of this study, different surfactants were tested regarding their effect on the dissolution of lignin (kraft lignin selected as a “model” lignin). As can be observed in Figure 3, cationic surfactants strongly interact with lignin, thus impacting the dissolution efficiency. Two striking observations can be made: (1) the dissolution performance remarkably increases above the critical aggregation concentration (CAC) of the surfactant, and (2) longer aliphatic chains induce a superior interaction between lignin and the surfactants, resulting in a lower CAC value [33,34]. Tetradecyltrimethylammonium bromide was observed to have a stronger affinity with lignin and its effect on the dissolution efficiency starts at lower concentrations. Note that the CMC of TTAB (0.00386 mol L^−1^ [35]) is lower than DeTAB, while OTAB shows the highest CMC (0.22 mol L^−1^ [36]) because of its less pronounced hydrophobicity. This effect demonstrates the important role of hydrophobic interactions in the surfactant–lignin complex formation. The pronounced beneficial effect of cationic surfactants in lignin dissolution is mainly driven by the favorable electrostatic interactions between the positively charged surfactant headgroups and the ionized carbonyl groups of lignin [17]. The high electrostatic attraction has also been found to be beneficial in other applications, such as in dye adsorption [37].

**Figure 4:**
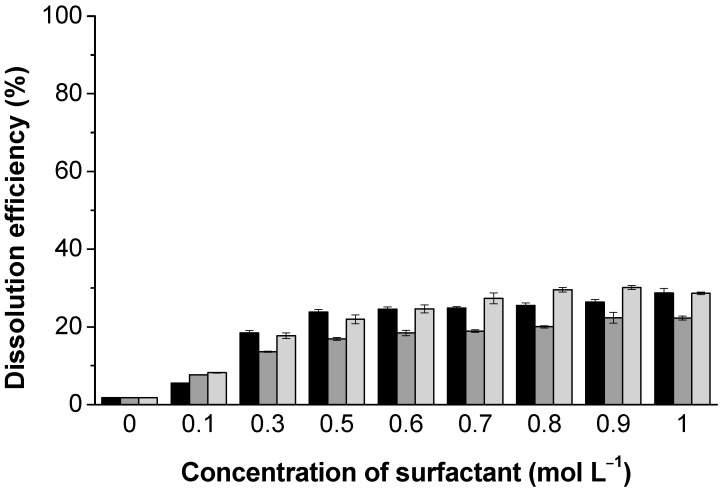
Dissolution efficiency of kraft lignin in water as a function of the anionic surfactants sodium octyl sulfate (SOS) (black), sodium decyl sulfate (SDeS) (gray), and sodium dodecyl sulfate (SDS) (light gray).

In contrast to cationic surfactants, anionic surfactants have a modest effect on dissolution. Regardless of the anionic surfactant used (i.e., SOS, SDeS, and SDS), the lignin dissolution is weakly affected and rather similar; at a surfactant concentration range between 0.1 and 0.5 mol L^−1^ (well above the CMCs), the dissolution efficiency is observed to increase to 24%, while above this concentration (i.e., from 0.5 to 1.0 mol L^−1^) their effect is very subtle—only a 4% improvement is observed (Figure 4). It is worth mention that the obtained solutions have an average pH slightly higher (5.30–5.50) than the pKa of carbonyl groups (pKa 4–5 [38]). Consequently, electrostatic repulsion between the ionized carbonyl groups of lignin and the ionized sulfate groups of the anionic surfactants may limit their interaction and eventual favorable effects on lignin dissolution. On the other hand, such deprotonation of lignin carbonyl groups may give rise to the stretching of the lignin structure, inducing the increase in its solubility [39]. Another possibility for rationalizing the moderate increase in the lignin dissolution is related to the increase in counter-ion condensation, both on the surfactant and lignin, as suggested elsewhere [40].

**Figure 5:**
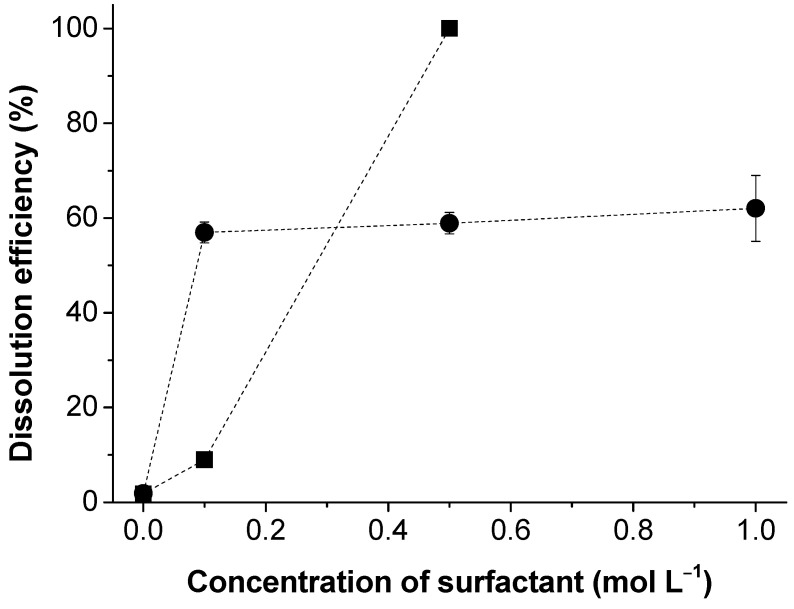
Dissolution efficiency of kraft lignin as a function of polysorbate 20 (PS20) (squares), and Triton X-100 (TX-100) (circles).

To better understand the role of ionic interactions on the lignin dissolution mechanism, nonionic surfactants were also tested. Two different non-ionic surfactants with different molecular weights and CMCs, PS20 (CMC = 0.0489 mmol L^−1^ [41]) and TX-100 (CMC = 0.240 mmol L^−1^ [41]), were used at concentrations well above their CMCs. As it can be observed in Figure 5, both non-ionic surfactants show a notable beneficial effect on the lignin dissolution. Nevertheless, whilst the TX-100 reaches a dissolution efficiency of ca. 60% and becomes concentration-independent, the PS20 shows a sharp increase, between 0.1 and 0.5 mol L^−1^, reaching 100% dissolution efficiency for the higher concentrations. The reason for the observed behavior can be related to the relative number of ethylene and hydroxyl groups in TX-100 and PS20, and the occurrence of hydrogen bonds between the ethylene oxide and hydroxyl units from the surfactants and the hydroxyl groups of the lignin. Those interactions give rise to the formation of lignin–surfactant complexes, similar to those described for guar gum [42]. Lignin contains both hydrogen bond donors and acceptor groups, and it is thus capable of forming intra- and intermolecular hydrogen bonds. The presence of the nonionic surfactants may compete and/or break some of the intra- and intermolecular hydrogen bonds among lignin molecules, driving its dissolution [43].

**Figure 6:**
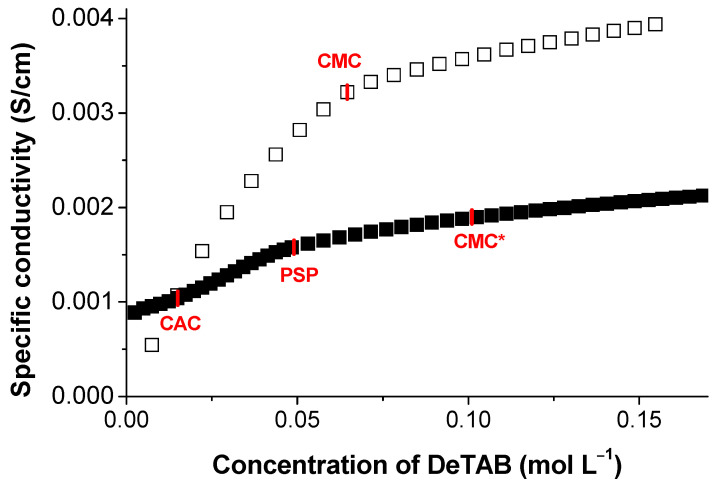
Specific conductivity of DeTAB at different concentrations in water (empty squares) and in an aqueous solution of 10 wt.% kraft lignin (full squares). The different transition regions highlighted (i.e., critical aggregation concentration (CAC), polymer saturation point (PSP), critical micelle concentration (CMC), and apparent critical micelle concentration (CMC*)) are described in the text.

Several techniques have been used to study the interaction between different polymers and surfactants, such as small-angle neutron scattering [44], fluorescence [45], specific conductivity [46], dynamic light scattering [47], surface tension [48], NMR spectroscopy [49,50], and rheometry [42,51]. To elucidate the dissolution mechanism, the surfactant–lignin interactions were further evaluated by conductimetry. It is important to emphasize that electrical conductivity is an important tool to unravel polymer–surfactant interactions [52]. Among the tested surfactants, the cationic DeTAB was selected due to its striking beneficial effect (see Figure 3) and availability. In Figure 6, the electrical conductance of DeTAB in water, in the presence and absence of lignin, is represented. The variation in κ with the concentration of DeTAB shows an expected profile with two linear regions, at pre- and post-CMC regions, with an estimated CMC of 0.065 (± 0.005) mol L^−1^, in agreement with the literature [53,54]. In the presence of 10 wt.% kraft lignin, the conductometric behavior of DeTAB changes significantly, and two remarks are worth noting: 1) the electrical conductivity is lower than in the absence of lignin, which may be related to an increase in the viscosity or due to the surfactant–polymer attractive interactions inducing some charge neutralization (this is consistent with the decrease in the slope of κ=*f*([DeTAB]) [55], and 2) the dependence of κ with[DeTAB] shows three inflection points rationalized as follows. The first one, occurring at a DeTAB concentration around 0.015 mol L^−1^, can be assigned to the CAC [56] relative to polymer-free micelle formation, which marks the onset of the association between the polymer and the surfactant; the second inflection point corresponds to the polymer saturation point (PSP), attributed to the surfactant concentration needed to saturate the polymer chains [34,57,58,59]. Between CAC and PSP, a dynamic equilibrium between surfactant-saturated polymer and micelles exists [52]. Above the PSP, the surfactant in excess forms regular free micelles, in dynamic equilibrium with the polymer–surfactant complexes [57]. This is in line with a third inflection point, which is justified by the micelle formation and corresponds to the so-called “apparent critical micelle concentration” (CMC*) of the DeTAB. The effect of lignin concentration on the micellization properties of DeTAB is shown in Table 1. The CAC values decrease with lignin concentration since the association between lignin and the surfactant becomes more favorable [59]. On the other hand, the PSP values increase with lignin concentration because of the higher polymer chains/surfactant micelles ratio. This phenomenon has been observed in other systems [34,58,59]. It can also be seen that the formation of surfactant micelles, at concentrations above the CMC, cannot be justified on thermodynamic grounds. However, once the polymer favors the interaction with the surfactant, it should be expected that surfactants consumed in the interaction are subtracted from the CMC*; consequently, the effective CMC of DeTAB in the presence of lignin should be calculated as CMC’ = CMC*- PSP (see Table 1). A deeper analysis of the effect of the DeTAB micellization can be done based on the standard Gibbs energy of micellization, Δ*G*^0^*_m_*

(1) $$\Delta G_{m}^{0}=\left(2-\alpha \right)RTlnX$$

where *R* is the gas constant and *X* is the mole fraction of the surfactant at the CMC. The variable α represents the degree of dissociation of counterions which, in the present case, is computed as the slope of the electrical conductance data as a function of surfactant concentration, at the post-micelle region, and pre-CAC region, following the recommendations of Zanette et al. [60]. Based on the values reported in Table 1, and although the data are somewhat scattered, it is possible to conclude that lignin, at the lowest concentration, leads to the formation of more stable DeTAB micelles, with the Δ*G*^0^*_m_* ca. 14% lower than that obtained for the lignin-free micellization of DeTAB; this is also driven by a very low α, suggesting the occurrence of highly dense micelles in the presence of lignin. This result agrees with the highest solubility found for this lignin concentration.

### 3.2. Lignin Extraction

**Figure 7:**
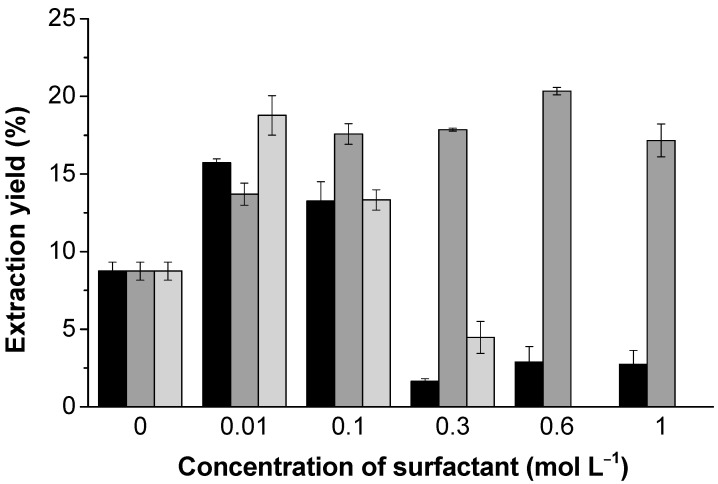
Yield of the recovered lignin using different concentrations of SDS (black), hexadecyltrimethylammonium bromide (CTAB) (gray), and PS20 (light gray).

In the first part of this study, several surfactants were tested regarding their effect on lignin dissolution. Among them, the non-ionic PS20, cationic CTAB, and the anionic SDS were selected and used as additives to improve the lignin extraction from pine sawdust. The solvent used was formic acid (98–100%) [61], to guarantee that the phenolic hydroxyl and carbonyl groups are protonated. Note that CTAB was selected for the extraction trials due to the higher availability and solubility of this surfactant in the acidic solvent. As can be observed in Figure 7, the SDS and PS20 have a positive effect on the extraction yield at lower concentrations (i.e., at concentrations lower than 0.1 mol L^−1^). Higher amounts of these surfactants have a negative effect on the extraction yield, due to the presence of micelles. On the other hand, the addition of CTAB in a concentration range from 0.01 to 1 mol L^−1^ leads to an enhanced lignin extraction; the extraction yield has a threefold increase when the concentration increases from 0 to 0.6 mmol L^−1^. Nevertheless, the increase in surfactant concentration favors the extraction of impurities, such as sugars [62,63], proteins, and minerals [64]. This is verified by the gradual decrease in lignin purity, from 87 (± 1) to 61 (± 2) %, using 0.01 and 1 mol L^−1^, respectively. The same trend was observed using SDS and PS20, with the lignin purity decreasing from 80.1 (± 0.9) to 72 (± 1) % for SDS and from 79 (± 1) and 73.4 (± 0.6) % for PS20, using 0.01 and 0.1 mol L^−1^ of each type of surfactant, respectively. In previous studies, using a related acid solvent (H_2_SO_4_ (aq.)), the addition of sodium dodecylbenzene sulfonate (SDBS), tween 80, and dodecylbenzene sulfonic acid (DDBSA) were also observed to increase the lignin extraction from biomass [14,15]. Hamzeh et al. used non-ionic surfactants in soda pulping and verified that, in most cases, increasing amounts of surfactants decreases pulping yields and delignification rate [16]. It is suggested that the prevalent forces are hydrophobic and π –π interactions driving the lignin molecular backbone to contract and clamp adsorbed the surfactant [39]. The addition of surfactants improves penetration efficiency through wetting and emulsifying-like effects [65,66].

**Figure 8:**
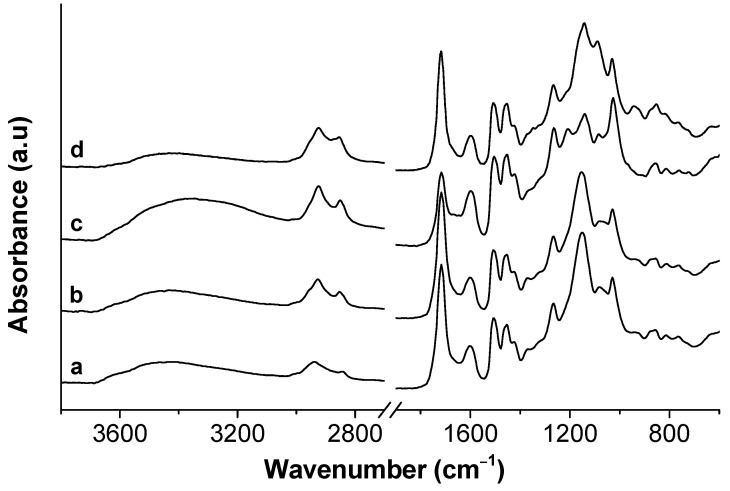
Normalized FTIR spectra of lignin obtained after extraction with (a) formic acid and 0.01 mol L^−1^ of (b) SDS, (c) CTAB, and (d) PS20.

The lignins recovered using 0.01 mol L^−1^ of surfactant were analyzed by FTIR and TG, and the results compared with lignin recovered without surfactant. Characteristic bands of lignin were found in all extracted lignins (Figure 8): O-H vibrations (3200–3500 cm^−1^) [67]; CH stretching in aromatic methoxyl or methylene groups of side chain (2925–2940 cm^−1^) [68]; CH vibration of -OCH_3_ groups (2840–2870 cm^−1^) [68]; C=O stretching in unconjugated ketone, carbonyl, and in ester groups (1716 cm^−1^) [69]; aromatic skeletal vibrations plus the C=O stretch (1600 cm^−1^) [69]; aromatic skeleton vibration (1506 cm^−1^) [69]; C-H bending from methyl or methylene groups (1455 cm^−1^) [68]; aromatic skeletal vibration combined with CH asymmetric deformation of methyl groups (1423 cm^−1^) [69]; guaiacyl ring breathing with CO stretch in lignin; CO linkage in guaiacyl aromatic methoxyl groups (1266 cm^−1^) [68]; aromatic C-H in plane deformation plus secondary alcohols plus C=O stretch (1142–1152 cm^−1^); C-O deformation in secondary alcohol and aliphatic ethers (1082–1088 cm^−1^) [69]; aromatic C-H in-plane deformation plus C-O deformation in primary alcohols; and C=O stretch (unconjugated) (1029 cm^−1^) [69].

Depending on the solvent and operational conditions used in the extraction, the lignin structure may differ. In this case, the spectrum of extracted lignin using SDS as an additive is very similar to the one of lignin extracted with only formic acid (surfactant-free). Using CTAB and TX-100, it is possible to observe some changes in the relative absorbance in the characteristic bands of lignin.

**Figure 9:**
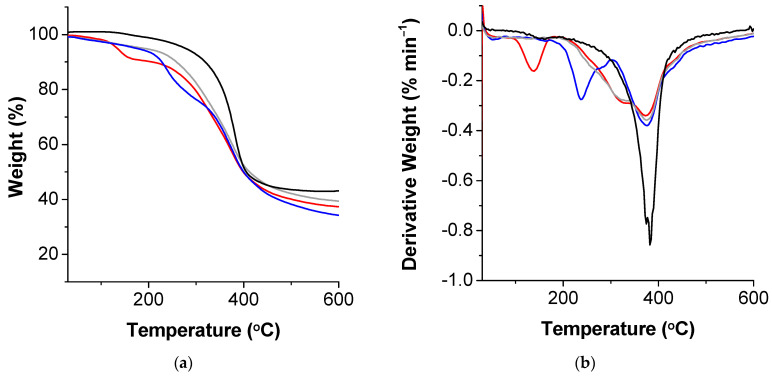
(**a**) Thermograms and (**b**) the corresponding differential thermograms (DTGs) of recovered lignin from extraction with formic acid (gray line), and 0.01 mol L^−1^ of SDS (red line), CTAB (blue line), and PS20 (black line).

The thermal decomposition profiles of the recovered lignins are shown in Figure 9. Using SDS, the extracted lignin has a peak at 137 °C, due to the volatilization of low-molecular-weight compounds in addition to removal of water [67]. This value shifts to 163 °C for the lignin obtained with the other surfactants and the intensity is much lower. Using CTAB, the lignin obtained contains hemicellulose impurities, due to the peak at 237 °C [70]. All lignins show a temperature at the maximum rate of weight loss between 373 and 382 °C, due to the fragmentation of inter-unit linkages [71]. These values are in accordance with those found for other lignins (from 300 to 450 °C) [70]. The larger proportion of weight loss between 200 and 600 °C, observed in lignin recovered without surfactant and with SDS, can be attributed to the presence of carbohydrates [72].

## 4. Conclusions

In this study, a systematic analysis on the effect of different surfactants on the dissolution of model kraft lignin and their influence on lignin extraction from lignocellulose waste (maritime pine sawdust) was conducted. Overall, surfactants proved to be remarkable additives to enhance kraft lignin dissolution and boost lignin extraction from biomass. Using cationic amphiphiles, the dissolution efficiency is proportional to the concentration of surfactant, being possible to fully dissolve the initial amount of lignin (i.e., 20 wt.%). A much less significant effect was observed when using anionic surfactants with a maximum dissolution efficiency of ca. 30% (0.9 mol L^−1^ SDS). With non-ionic surfactants, it was possible to completely dissolve lignin using 0.5 mol L^−1^ PS20. The data suggests that the hydrophobic interactions play an important role in the surfactant–lignin complex formation. This effect is boosted by the favorable electrostatic interactions between the cationic surfactants and ionized lignin, while it is decreased when anionic surfactants are used due to the repulsion between likewise charges. At low surfactant concentrations (i.e., 0.01 and 0.1 mol L^−1^), all surfactants studied are observed to have a positive effect on lignin extraction from biomass. The increase in surfactant concentration only proved to be beneficial in the extracted lignin yield when using CTAB. Nevertheless, the higher extraction yield is also followed by a growing number of impurities. Therefore, this implies a fine-tune control to strictly balance the yield and purity regarding the hypothetical final applications. This work not only introduces the usefulness of surfactants as valuable additives in lignin dissolution and extraction but also sheds light on the mechanisms of dissolution/extraction and the concomitant role of electrostatic and hydrophobic interactions. This knowledge is of critical importance for the future development of more efficient dissolution/extraction strategies, thus paving the way for lignin valorization.

## Figures and Tables

**Table 1:** The CMC, CAC, and PSP of DeTAB for different initial concentrations of lignin obtained from the conductivity data at 25 °C.

Kraft Lignin (wt.%)	CAC (mol L^−1^)	PSP (mol L^−1^)	CMC’ (mmol L^−1^)	Δ*G*^0^*_m_* (kJ mol^−1^)
0.0	0.065 ± 0.005 *	-	64.6	−29.8
0.1	0.020 ± 0.002	0.036 ± 0.004	48.0	−33.9
1.0	0.020 ± 0.002	0.040 ± 0.006	70.0	−31.2
2.5	0.018 ± 0.002	0.039 ± 0.006	52.0	−32.5
5.0	0.018 ± 0.002	0.041 ± 0.007	72.0	−30.9
10.0	0.015 ± 0.002	0.049 ± 0.002	53.0	−28.9

* This value corresponds to the CMC of DeTAB in the absence of lignin.

## Data Availability

The data presented in this study are available in this article.

## References

[1] Goddard E.D., Ananthapadmanabhan K.P. (2018). Interactions of Surfactants with Polymers and Proteins.

[2] Kwak J.C.T. (2020). Polymer-Surfactant Systems.

[3] Kronberg B., Lindman B. (2003). Surfactants and Polymers in Aqueous Solution.

[4] Kronberg B., Holmberg K., Lindman B. (2014). Surface Chemistry of Surfactants and Polymers.

[5] Kai D., Tan M.J., Chee P.L., Chua Y.K., Yap Y.L., Loh X.J. (2016). Towards lignin-based functional materials in a sustainable world. Green Chem..

[6] Zhang L., Gellerstedt G. (2001). NMR observation of a new lignin structure, a spiro-dienone. Chem. Commun..

[7] Melro E., Alves L., Antunes F.E., Medronho B. (2018). A brief overview on lignin dissolution. J. Mol. Liq..

[8] Jiang F., Qian C., Esker A.R., Roman M. (2017). Effect of Nonionic Surfactants on Dispersion and Polar Interactions in the Adsorption of Cellulases onto Lignin. J. Phys. Chem. B.

[9] Ren B., Gao Y., Lu L., Liu X., Tong Z. (2006). Aggregates of alginates binding with surfactants of single and twin alkyl chains in aqueous solutions: Fluorescence and dynamic light scattering studies. Carbohydr. Polym..

[10] Xu A., Li W., Zhang Y., Xu H. (2016). Eco-friendly polysorbate aqueous solvents for efficient dissolution of lignin. RSC Adv..

[11] Norgren M., Edlund H. (2001). Stabilisation of kraft lignin solutions by surfactant additions. Colloids Surf. Physicochem. Eng. Asp..

[12] Singh S.K. (2020). Biological treatment of plant biomass and factors affecting bioactivity. J. Clean. Prod..

[13] Zheng Y., Guo M., Zhou Q., Liu H. (2019). Effect of lignin degradation product sinapyl alcohol on laccase catalysis during lignin degradation. Ind. Crops Prod..

[14] Wang B., Yang G., Wang Q., Liu S., Chen J., Fang G. (2020). A new surfactant assisted acid prehydrolysis process for enhancing biomass pretreatment. Cellulose.

[15] Qing Q., Yang B., Wyman C.E. (2010). Impact of surfactants on pretreatment of corn stover. Bioresour. Technol..

[16] Hamzeh Y., Abyaz A., Niaraki M.O.S.M., Abdulkhani A. (2009). Application of surfactants as pulping additives in soda pulping of bagasse. BioResources.

[17] Norgren M., Mackin S. (2009). Sulfate and surfactants as boosters of kraft lignin precipitation. Ind. Eng. Chem. Res..

[18] Wang W., Zhuang X., Tan X., Wang Q., Chen X., Yu Q., Qi W., Wang Z., Yuan Z. (2018). Dual Effect of Nonionic Surfactants on Improving the Enzymatic Hydrolysis of Lignocellulose. Energy Fuels.

[19] Mesquita J.F., Ferraz A., Aguiar A. (2016). Alkaline-sulfite pretreatment and use of surfactants during enzymatic hydrolysis to enhance ethanol production from sugarcane bagasse. Bioprocess. Biosyst. Eng..

[20] Zhang H., Lyu G., Zhang A., Li X., Xie J. (2018). Effects of ferric chloride pretreatment and surfactants on the sugar production from sugarcane bagasse. Bioresour. Technol..

[21] Qi B., Chen X., Wan Y. (2010). Pretreatment of wheat straw by nonionic surfactant-assisted dilute acid for enhancing enzymatic hydrolysis and ethanol production. Bioresour. Technol..

[22] Parnthong J., Kungsanant S., Chavadej S. (2018). The Influence of Nonionic Surfactant Adsorption on Enzymatic Hydrolysis of Oil Palm Fruit Bunch. Appl. Biochem. Biotechnol..

[23] Alkasrawi M., Eriksson T., Börjesson J., Wingren A., Galbe M., Tjerneld F., Zacchi G. (2003). The effect of Tween-20 on simultaneous saccharification and fermentation of softwood to ethanol. Enzyme Microb. Technol..

[24] Li K., Wan J., Wang X., Wang J., Zhang J. (2016). Comparison of dilute acid and alkali pretreatments in production of fermentable sugars from bamboo: Effect of Tween 80. Ind. Crops Prod..

[25] Chen C., Jiang L., Ma G., Jin D., Zhao L., Ouyang X. (2019). Lignin Removal from Tobacco Stem with Laccase Improved by Synergistic Action of Weak Alkali and Tween 80. Waste Biomass Valorization.

[26] Zheng Y., Pan Z., Zhang R., Wang D., Jenkins B. (2007). Non-Ionic Surfactants and Non-Catalytic Protein Treatment on Enzymatic Hydrolysis of Pretreated Creeping Wild Ryegrass.

[27] Méndez Arias J., de Oliveira Moraes A., Modesto L.F.A., de Castro A.M., Pereira N. (2017). Addition of Surfactants and Non-Hydrolytic Proteins and Their Influence on Enzymatic Hydrolysis of Pretreated Sugarcane Bagasse. Appl. Biochem. Biotechnol..

[28] Chen Y.A., Zhou Y., Liu D., Zhao X., Qin Y. (2018). Evaluation of the action of Tween 20 non-ionic surfactant during enzymatic hydrolysis of lignocellulose: Pretreatment, hydrolysis conditions and lignin structure. Bioresour. Technol..

[29] Kim H.J., Kim S.B., Kim C.J. (2007). The effects of nonionic surfactants on the pretreatment and enzymatic hydrolysis of recycled newspaper. Biotechnol. Bioprocess. Eng..

[30] Barthel J., Feuerlein F., Neueder R., Wachter R. (1980). Calibration of conductance cells at various temperatures. J. Solut. Chem..

[31] Templeton D., Ehrman T. (1995). Chemical Analysis and Testing Task: LAP-003 (Determination of Acid-Insoluble Lignin in Biomass).

[32] Ehrman T. (1996). Determination of Acid Soluble Lignin in Biomass, Chemical Analysis and Testing Task, Laboratory Analytical Procedure (LAP-004).

[33] Ansari A.A., Kamil M., Kabir-ud-Din (2013). Polymer-Surfactant Interactions and the Effect of Tail Size Variation on Micellization Process of Cationic ATAB Surfactants in Aqueous Medium. J. Dispers. Sci. Technol..

[34] Banipal T.S., Kaur H., Banipal P.K., Sood A.K. (2014). Effect of head groups, temperature, and polymer concentration on surfactant—Polymer interactions. J. Surfactants Deterg..

[35] Ruiz C.C. (1999). Thermodynamics of micellization of tetradecyltrimethylammonium bromide in ethylene glycol-water binary mixtures. Colloid Polym. Sci..

[36] Jang J., Bae J., Park E. (2006). Selective fabrication of poly(3,4-ethylenedioxythiophene) nanocapsules and mesocellular foams using surfactant-mediated interfacial polymerization. Adv. Mater..

[37] Albadarin A.B., Collins M.N., Naushad M., Shirazian S., Walker G., Mangwandi C. (2017). Activated lignin-chitosan extruded blends for efficient adsorption of methylene blue. Chem. Eng. J..

[38] Notley S.M., Norgren M. (2008). Adsorption of a strong polyelectrolyte to model lignin surfaces. Biomacromolecules.

[39] Pang Y., Wang S., Qiu X., Luo Y., Lou H., Huang J. (2017). Preparation of Lignin/Sodium Dodecyl Sulfate Composite Nanoparticles and Their Application in Pickering Emulsion Template-Based Microencapsulation. J. Agric. Food Chem..

[40] Notley S.M., Norgren M. (2006). Measurement of interaction forces between lignin and cellulose as a function of aqueous electrolyte solution conditions. Langmuir.

[41] Helenius A., Simons K. (1975). Solubilization of membranes by detergents. BBA Rev. Biomembr..

[42] Fijan R., Šostar-Turk S., Lapasin R. (2007). Rheological study of interactions between non-ionic surfactants and polysaccharide thickeners used in textile printing. Carbohydr. Polym..

[43] Wang J., Li Y., Qiu X., Liu D., Yang D., Liu W., Qian Y. (2017). Dissolution of lignin in green urea aqueous solution. Appl. Surf. Sci..

[44] Appell J., Porte G., Rawiso M. (1998). Interactions between Nonionic Surfactant Micelles Introduced by a Telechelic Polymer. A Small Angle Neutron Scattering Study. Langmuir.

[45] Winnik F.M., Regismond S.T.A. (1996). Fluorescence methods in the study of the interactions of surfactants with polymers. Colloids Surf. Physicochem. Eng. Asp..

[46] Bakshi M.S., Sachar S. (2004). Surfactant polymer interactions between strongly interacting cationic surfactants and anionic polyelectrolytes from conductivity and turbidity measurements. Colloid Polym. Sci..

[47] Ali M.S., Suhail M., Ghosh G., Kamil M., Kabir-ud-Din (2009). Interactions between cationic gemini/conventional surfactants with polyvinylpyrrolidone: Specific conductivity and dynamic light scattering studies. Colloids Surf. Physicochem. Eng. Asp..

[48] Kaur R., Kumar S., Aswal V.K., Mahajan R.K. (2012). Interactional and aggregation behavior of twin tail cationic surfactants with pluronic L64 in aqueous solution. Colloid Polym. Sci..

[49] Cabane B. (1977). Structure of some polymer-detergent aggregates in water. J. Phys. Chem..

[50] Pettersson E., Topgaard D., Stilbs P., Söderman O. (2004). Surfactant/Nonionic Polymer Interaction. A NMR Diffusometry and NMR Electrophoretic Investigation. Langmuir.

[51] Rivero D., Gouveia L.M., Müller A.J., Sáez A.E. (2012). Shear-thickening behavior of high molecular weight poly(ethylene oxide) solutions. Rheol. Acta.

[52] Khan M.Y., Samanta A., Ojha K., Mandal A. (2008). Interaction between aqueous solutions of polymer and surfactant and its effect on physicochemical properties. Asia-Pac. J. Chem. Eng..

[53] Ribeiro A.C., Lobo V.M.M., Valente A.J.M., Azevedo E.F., Miguel M.D.G., Burrows H.D. (2004). Transport properties of alkyltrimethylammonium bromide surfactants in aqueous solutions. Colloid Polym. Sci..

[54] Evans D.F., Allen M., Ninham B.W., Fouda A. (1984). Critical micelle concentrations for alkyltrimethylammonium bromides in water from 25 to 160 °C. J. Solut. Chem..

[55] Burrows H.D., Valente A.J.M., Costa T., Stewart B., Tapia M.J., Scherf U. (2015). What conjugated polyelectrolytes tell us about aggregation in polyelectrolyte/surfactant systems. J. Mol. Liq..

[56] Diamant H., Andelman D. (1999). Onset of self-assembly in polymer-surfactant systems. Europhys. Lett..

[57] Dal-Bó A.G., Laus R., Felippe A.C., Zanette D., Minatti E. (2011). Association of anionic surfactant mixed micelles with hydrophobically modified ethyl(hydroxyethyl)cellulose. Colloids Surf. Physicochem. Eng. Asp..

[58] Sood A.K., Singh K., Banipal T.S. (2009). Study of micellization behavior of some alkyldimethylbenzyl ammonium chloride surfactants in the presence of polymers. J. Dispers. Sci. Technol..

[59] Silva S.M.C., Antunes F.E., Sousa J.J.S., Valente A.J.M., Pais A.A.C.C. (2011). New insights on the interaction between hydroxypropylmethyl cellulose and sodium dodecyl sulfate. Carbohydr. Polym..

[60] Zanette D., Ruzza Â.A., Froehner S.J., Minatti E. (1996). Polymer-surfactant interactions demonstrated by a kinetic probe: Degree of ionization. Colloids Surf. Physicochem. Eng. Asp..

[61] Zhang M., Qi W., Liu R., Su R., Wu S., He Z. (2010). Fractionating lignocellulose by formic acid: Characterization of major components. Biomass Bioenergy.

[62] Inkrod C., Raita M., Champreda V., Laosiripojana N. (2018). Characteristics of Lignin Extracted from Different Lignocellulosic Materials via Organosolv Fractionation. Bioenergy Res..

[63] Erdocia X., Prado R., Corcuera M.Á., Labidi J. (2014). Effect of different organosolv treatments on the structure and properties of olive tree pruning lignin. J. Ind. Eng. Chem..

[64] Snelders J., Dornez E., Benjelloun-Mlayah B., Huijgen W.J.J., de Wild P.J., Gosselink R.J.A., Gerritsma J., Courtin C.M. (2014). Biorefining of wheat straw using an acetic and formic acid based organosolv fractionation process. Bioresour. Technol..

[65] Malkov S., Tikka P., Gullichsen J. (2001). Towards complete impregnation of wood chips with aqueous solutions. Part I. A retrospective and critical evaluation of the penetration process. Pap. Puu/Paper Timber.

[66] Parthasarathy V.R., Grygotis R.C., Wahoske K.W., Bryer D.M. (1996). A sulfur-free, chlorine-free alternative to kraft pulping. Tappi J..

[67] Rashid T., Kait C.F., Regupathi I., Murugesan T. (2016). Dissolution of kraft lignin using Protic Ionic Liquids and characterization. Ind. Crops Prod..

[68] Sathawong S., Sridach W., Techato K.A. (2018). Lignin: Isolation and preparing the lignin based hydrogel. J. Environ. Chem. Eng..

[69] Faix O. (1991). Classification of Lignins from Different Botanical Origins by FT-IR Spectroscopy. Holzforschung.

[70] Sun R.C., Tomkinson J., Lloyd Jones G. (2000). Fractional characterization of ash-AQ lignin by successive extraction with organic solvents from oil palm EFB fibre. Polym. Degrad. Stab..

[71] Tejado A., Peña C., Labidi J., Echeverria J.M., Mondragon I. (2007). Physico-chemical characterization of lignins from different sources for use in phenol-formaldehyde resin synthesis. Bioresour. Technol..

[72] Sameni J., Krigstin S., dos Santos Rosa D., Leao A., Sain M. (2014). Thermal characteristics of lignin residue from industrial processes. BioResources.

